# Efficacy of a Workplace Intervention Program With Web-Based Online and Offline Modalities for Improving Workers' Mental Health

**DOI:** 10.3389/fpsyt.2022.888157

**Published:** 2022-05-31

**Authors:** Lawrence T. Lam, Mary K. Lam, Prasuna Reddy, Prudence Wong

**Affiliations:** ^1^Eastern Health Clinical School, Monash University, Melbourne, VIC, Australia; ^2^Faculty of Health, University of Technology Sydney, Sydney, NSW, Australia; ^3^School of Health and Biomedical Sciences, RMIT University, Melbourne, VIC, Australia; ^4^Faculty of Science & Technology, Swinburne University of Technology, Melbourne, VIC, Australia; ^5^The Mental Health Association Hong Kong, Kowloon, Hong Kong SAR, China

**Keywords:** work-related burnout, stress, mental health literacy, randomized controlled trial, psychoeducation, web-based intervention

## Abstract

**Objective:**

This study aims to examine the efficacy of the Workplace Web-based blended psychoeducation mental health intervention program. Of particular interest is the short-term effect of the intervention on workplace burnout, stress, quality of life, and the mental health literacy of workers.

**Methods and Materials:**

The study focused on employees (*n* = 456) in specific industries with high levels of work-related stress, adopting a phase III wait-listed cluster randomized controlled trial. Work-related burnout was assessed by the Maslach Burnout Inventory (MBI) and stress was measured using the stress subscale of the Depression, Anxiety, and Stress scale (DASS). Quality of Life was evaluated by the European Quality of Life-5 Dimensions (EQ-5D-5L) and Mental Health Literacy was assessed using the Australian National Mental Health Literacy and Stigma Survey. Data were analyzed as a trial with intention-to-treat analysis and adjustment for the clustering effect of work sites.

**Results:**

Significant differences between intervention and control groups were found on all outcome measures except the self-rated quality of life. The intervention group displayed a significant reduction in the weighted mean score of about 1.0 units (s.e. = 0.4) on the stress scale (*p* = 0.015) and an increase in the weighted mean score of 1.9 units (s.e. = 0.9) in the professional accomplishment domain of the MBI (*p* = 0.035). Significant increases were found in the weighted mean scores in the intervention group for correct recognition of the mental problems, help-seeking, and stigmatization, in comparison to the control group who scored 0.2 (s.e. = 0.1), 0.9 (s.e. = 0.2), 1.8 (s.e. = 0.4), respectively.

**Conclusions:**

The results obtained from a comparison of the outcome measures between the intervention and control groups were statistically significant, indicating that the intervention group performed better on most measures. The study demonstrates that, in the short term. the on-and-offline modalities of the Web-based blended psychoeducation intervention program is efficacious in reducing workplace burnout and stress and promoting mental health literacy at the workplace.

## Introduction

Work is an important part of daily life for many people. The Organization for Economic Co-operation and Development (OECD) has estimated that, on average, full-time workers in the OECD countries spent 37% of their time working on a normal day (1). Workplace mental health has long been one of the major health concerns globally. The workplace is also an important venue for preventing mental health problems and for promoting mental wellness (2). The World Health Organization (WHO) has also stated in its comprehensive mental health action plan 2013–2020 that the workplace should be the main focal environment for mental health promotion (3). The plan emphasized that community-based, including the workplace, prevention and early intervention of mental health problems is of paramount importance and the improvement of the mental wellness of the working population should be considered a priority (3). Programs specifically designed for employees in the workplace could provide great benefits in terms of early identification and intervention of mental health problems.

As burnout and stress in the workplace are common precursors of mental health problems, the majority of intervention programs developed in the past were mainly designed to address these issues (4–7). Burnout is classified as an occupational phenomenon, not as a medical condition, in the 11th version of the International Classification of Diseases (ICD-11). It is defined as: “a syndrome conceptualized as resulting from chronic workplace stress that has not been successfully managed.” (8) In terms of the focus of intervention, there would be multiple points of attack and thus different approaches. Awa et al. reviewed different intervention programs for the effectiveness of reducing burnout and concluded that there were mainly two types of intervention programs, either person-directed or organization-directed (4). The effectiveness of person-directed intervention programs seemed to last for a short term of 6 months or less, programs that were directed at the organization level demonstrated an effect that could last for 12 months or longer. The researchers proposed that dual foci programs directed at the organization and the individual should be developed for future workplace intervention (4).

Psychoeducation is defined as “an intervention with systematic, structured, and didactic knowledge transfer for an illness and its treatment, integrating emotional and motivational aspects to enable patients to cope with the illness and to improve its treatment adherence and efficacy” by Ekhtiari et al. (9). It has been identified as an effective intervention strategy for mental health and has long been adopted in different populations (10, 11). For example, a randomized controlled study conducted by Shin and Lukens demonstrated the efficacy of a culturally sensitive psychoeducation intervention program for chronic mental illness (12). In addition to individual supportive therapy, psychoeducation was significantly more effective in terms of mental health outcomes than individual supportive therapy alone (12). With the advancement of information technologies, online or digital psychoeducation intervention offers the convenience of reaching the target audience more easily with greater acceptance. Psychoeducation, if supported by other measures such as supervision, may offer a better outcome (13). Online psychoeducation would also provide the benefit of continual treatment and support to individuals experiencing mental health problems even in difficult times, such as the current COVID-19 pandemic when personal contacts are greatly disrupted (14). In terms of the efficacy of online psychoeducation intervention programs targeting the workplace, particularly with multiple foci on workers and the workplace environment, little has been reported in the literature.

Many advantages of using an online or digital means for the delivery of training or education programs have been advocated. These include flexibility of learning material delivery, more independent learning, self-pacing, and self-responsible (15). Some disadvantages of using online teaching and training as the sole means of education delivery have also been identified, particularly from the students' or learners' perspective. Dumford and Miller, utilizing data from a national survey of university students, found that students who had participated in online learning were less likely to learn collaboratively, with fewer interactions with their teachers, and less inclined to discuss with others when compared with those taking face-to-face classes (16). These disadvantages may have a considerable impact on the effectiveness of a psychoeducation program used as an intervention strategy for mental health problems because interaction with and support from others are important elements of the therapeutic regime. Hence, blended learning, combining the online delivery of the learning materials and face-to-face live instructions and interaction between learners and instructor, has been suggested as a better approach (17).

Taking into consideration the suggestion by Awa et al. (4), a workplace intervention program was designed by the study team aiming as a preventive strategy for mental health problems and for promoting better mental health in the workplace (The WPMHL program). More specifically the intervention program targets the individual and organizational factors, particularly in enhancing the protective factors, that might affect the mental health status of workers. Details of the intervention program have been provided in the published protocol for a phase III wait-listed cluster randomized controlled trial for enhancing mental wellbeing in the workplace (18). In brief, the program comprises two main components, an individual-directed psychoeducation course, and an organization-directed consultation. The individual-directed psychoeducation course is based on the evidence-based Workplace Mental Health First Aid (MHFA) training program with additional elements on stress reduction and burnout prevention (19). This psychoeducation course is a blended program, using the e-Learning approach, consisting of six self-paced Web-based online e-Learning modules with a face-to-face group session at the end of the course. These modules cover the common mental health problems encountered by adults in daily life and at the workplace. These include depression, anxiety, obsessive-compulsive disorders, post-traumatic disorder, and others. It also includes some specific materials on burnout prevention and stress reduction. In each module, basic information, such as signs, symptoms, and possible causes, on these problems are presented with case studies and related activities for enhancing the learning of participants. The online modules are delivered through a purpose-built Web-based platform specifically designed for this project. The group session provides an opportunity for interactions among participants and the trainer for clarification of queries and for participants to gain hands-on experience through practice. The main focus of the psychoeducation modules is to enhance the mental health literacy of workers. Mental health literacy (MHL) was defined as “knowledge and beliefs about mental disorders which aid their recognition, management or prevention” by Jorm (20). There are different components embedded in this construct. These include the ability to recognize specific disorders, knowledge, and attitudes toward help-seeking, stigmatization, and social distancing from sufferers of mental health problems. For the organization-directed component, a consultation session is provided to the manager of the department or unit by a senior social worker with expertise in workplace issues. The consultation is based on the assessment of a Workplace Environment scan, using a standard protocol and the Moos Work Environment Scale (WES) (21). This uniquely designed dual-foci program aims to address the needs of the individual and issues in the work environment at the organization level.

This study aims to examine the efficacy of the intervention program through a phase III wait-listed cluster randomized controlled trial. It attempts to seek the answer to the research question of whether the program would reduce work-related burnout and stress; increase the general health-related quality of life and mental health literacy of participants. It is hypothesized that workers undergoing the intervention program would have a reduced level of work-related burnout and stress and an increase in health-related quality of life and mental health literacy in comparison to the controls. Of interest to the current report is the short-term effect (i.e., post-intervention), while data on the follow-up phase are still forthcoming.

## Materials and Methods

The full trial protocol of phase III wait-listed cluster randomized controlled trial (CRCT) was published previously in 2019 (18). In summary, the study focused on specific industries that were considered to have a high level of work-related stress, such as the servicing and hospitality industries. Six large-scale corporates agreed to participate in the project and employees were recruited from these corporates through their corresponding Human Resources departments. Participants joined the study voluntarily without any intervention from the management of these companies. Since the scope of business of these corporates covered a wide range of different work natures, ranging from manual labor to senior executives, the recruited sample also represented a multitude of different work types. Institution ethics approval for the study was granted by the Human Research Ethics Committee of the Tung Wah College (Ethics Approval number: REC2018020). The trial was also registered with the Australian New Zealand Clinical Trials Registry (ANZCTR, Registration number: ACTRN12619000464167). The intervention phase of the trial commenced in March 2021 and was completed in December 2021 with the follow-up phases still ongoing at the time of manuscript submission.

As a cluster randomized trial, the office sites were the primary unit of randomization. Upon recruitment, participants were screened for their eligibility with the exclusion of those who had been exposed to similar psychoeducation training. For random allocation of sites, a list of participating office sites with some basic staffing information was obtained from the Human Resources Departments of these corporates. The randomization was conducted by a qualified statistician who was blinded to the process of recruitment and the ongoing outcome assessments. The central registry was responsible for generating the randomization tables and provided the information to the field staff right after the briefing session and baseline data collection *via* instant messaging. Once registered, participants completed the baseline data collection *via* an Internet-based online platform specifically designed for the study. Participants nested in different site offices were then allocated to the intervention or the wait-listed control groups randomly according to a randomization schedule generated by the central registry. Post-intervention data collection took place immediately after the completion of the online psychoeducation training course and at the end of the face-to-face session, also *via* the online platform. Participants were then followed for 3 months with another data collection using the same method.

The intervention program was briefly described in the introduction and full detail in the previous publication by the authors (18). The full program, including both the organizational and psychoeducation components, lasted for 3 months for each site. Interested readers can refer to the publication for more information. The outcome measures of the study included work-related burnout, stress, general health-related quality of life, and mental health literacy.

The Maslach Burnout Inventory (MBI) was used to assess the work-related burnout of participants (22, 23). There are three domains, reflected in the three subscales, of the MBI capturing three different aspects of the burnout phenomena. One of the subscales is Emotional Exhaustion which measures the feeling that one is exhausted emotionally by work. Depersonalization captures the state that the worker is impersonally responding to clients or recipients of one's service or care. The last subscale is Personal Accomplishment which assesses the extent of the worker's competence and achievement in service provision. The MBI has been fully validated, translated into many languages, and employed widely in many countries (24, 25). The psychometric properties of the MBI were also studied by Wickramasinghe et al. resulting in a three-factor model which fitted the data significantly better than other alternative models. The internal consistency for the subscales was high with Cronbach's alpha values of 0.84, 0.87, and 0.88, respectively (24). In this study, the subscale scores were used for analyses.

Stress was assessed by the stress subscale of the Depression, Anxiety, and Stress scale (DASS) (26). A fully validated and commonly used instrument, the DASS was designed for assessing stress, depressive symptoms, and anxiety in the general population. The psychometric properties of the scale were well-studied showing strong reliability and validity for both the English and the Chinese versions (27, 28). The stress subscale consists of 7 items with a 4-point Likert response scale ranging from 0 (never) to 3 (almost always) resulting in a total summative score from 0 to 21. A recent study demonstrated a moderately high internal consistency for the stress subscale with a Cronbach's alpha value of 0.79 (29). In this study, the total summative score of the stress subscale was used for analyses.

Health-Related Quality of Life (HRQoL) was evaluated using the five-level version of European Quality of Life-5 Dimensions (EQ-5D-5L) (30). The instrument has been widely used in many countries and the validity has been well-demonstrated and published. The instrument was also translated into the Chinese language and the Chinese version of the EQ-5D-5L was validated with a moderately high Cronbach's alpha value of 0.78 and a good test-retest reliability of an Intraclass Correlation of 0.777 (31). For analysis purposes, the total raw scores of the EQ-5D-5L were used.

Mental Health Literacy was assessed using the Australian National Mental Health Literacy and Stigma Survey (32). The instrument has been validated and widely used in many studies in different countries (33). Since the full survey is lengthy and comprises many domains with different independent constructs, the full survey instrument was not used. In consideration of the specific local context, some components of the instrument were selected and utilized in the study. These included correct identification of mental health problems, help-seeking for a mental health problem, stigmatization, and social distancing.

Information on potential confounding factors was also collected in case an adjustment for the effect of confounding was necessary. These variables included demographics, employment duration, resignation intentions, health conditions, and behaviors, such as drinking, smoking, physical activity, and sick leaves.

The statistical software program Stata V17.0 was used for conducting data analyses (34). The statistical analytical approaches employed were based on the following considerations: (1) participants were nested in different corporates, thus adjustment for the clustering effect of the dataset is necessary; (2) outcome measures were continuous variables and measured repeatedly at baseline and post-intervention; (3) data collected at the baseline on both groups mimicked a cross-sectional survey, thus an appropriate corresponding approach need to be applied. A two-stage procedure was employed for the analyses. First, for examining participants' characteristics and the randomness of the sample baseline data were described and comparisons between groups were conducted. Adopting the approach for analyzing cross-sectional cluster data, baseline data were pre-set with the Stata *svy* command and weighted with the inverse of the sample size in each site as the weighting factor. Descriptive analyses were conducted using frequencies and percentages or means and standard errors. Comparisons of proportions and means between groups were carried out using Chi-squared tests and simple linear regression modeling, respectively, with adjustment for the clustering effect. Any variables with significant differences between groups would suggest a potential confounder and include them in further subsequent analyses. For the inclusion of potential confounders, a *p* < 0.20 was used as the inclusion criteria. Second, for investigating the efficacy of the intervention program, comparisons of the mean scores of the outcome measures between the intervention and control groups after the intervention program adjusting for the clustering effect, and the baseline assessment of the outcome measures were conducted. The Generalized Linear Latent And Mixed Model (GLLAMM) approach was employed to test for any group differences. To handle any loss to follow-up, the main analyses were conducted according to the principle of Intention-to-Treat (ITT) and missing data in any variables were imputed using the multiple imputation approach with an assumption of Missing at Random (MAR) for all missing values. An initial data cleaning procedure had revealed that only a few variables had missing values <2% of the sample size. A type I error rate of 5% was adopted for the testing of hypotheses.

## Results

In total, 456 participants were recruited to the trial from five corporates with 229 (50.2%) randomly allocated to receive the intervention program first. All participants in the intervention group completed the Web-based online modules and the face-to-face session within the designated time from the commencement of the intervention. Baseline data and the post-intervention outcome assessments were conducted for both the intervention and control groups within a week of completion of the intervention. Table 1 summarized the descriptive statistics on the demographic, health and work-related variables, and the baseline assessments on the outcome measures, work-related burnout, stress, quality of life, and MHL. Results on the comparisons of these variables between the intervention and control groups were also presented. As shown, none of these comparisons was statistically significant suggesting that the randomization process was performed satisfactorily. It is also noted that the comparisons of two demographic variables, namely sex and education level, resulted in a significance level *p* < 0.20. By the selection criteria, these two variables were included in further analyses.

**Table 1:** Frequency (%) or mean (s.e.) of participants' demographic, health and work-related variables, and baseline assessment of outcome variables by groups and the results on comparisons (*N* = 456).

**Participants' characteristics**	**Control** **(*n* = 227)**	**Intervention** **(*n* = 229)**	**Results on comparison^a^**
**Demographics**			
Age (years)	40.5 (1.1)	40.9 (1.4)	*p* = 0.575
Male sex	97 (42.7%)	118 (51.5%)	*p* = 0.104
Education level (University or above)	173 (76.2%)	190 (83.0%)	*p* = 0.109
Marital status (married)	135 (59.5%)	136 (59.4%)	*p* = 0.988
Full-time employment (yes)	227 (100.0%)	227 (99.1%)	*p* = 0.407
Flexible hours (yes)	82 (36.1%)	82 (35.8%)	*p* = 0.926
**Health and work-related variables**			
Regular exercise (yes)	178 (79.5%)	166 (73.8%)	*p* = 0.198
Smoker (yes)	8 (3.6%)	9 (4.0%)	*p* = 0.864
Drinker (moderate/heavy)	4 (1.8%)	4 (1.8%)	*p* = 0.981
Intended to resign (yes)	99 (43.6%)	107 (46.7%)	*p* = 0.203
**Outcome variables**			
Burnout — emotional exhaustion	20.1 (0.8)	20.9 (0.8)	*p* = 0.611
Burnout — depersonalization	6.5 (0.4)	6.6 (0.4)	*p* = 0.897
Burnout — professional accomplishment	28.0 (1.0)	28.0 (0.8)	*p* = 0.999
Stress	7.1 (0.3)	7.4 (0.3)	*p* = 0.541
Quality of life (self-rating)	80.7 (1.4)	70.3 (1.0)	*p* = 0.568
MHL — correct recognition	3.3 (0.05)	3.3 (0.03)	*p* = 0.567
MHL — help-seeking	12.1 (0.2)	12.2 (0.3)	*p* = 0.611
MHL — stigmatization	24.5 (0.5)	24.9 (0.5)	*p* = 0.213
MHL — distancing	12.1 (0.3)	12.2 (0.4)	*p* = 0.709

a *Adjusted for the clustering effect*.

Results of the comparisons of the post-intervention outcome measures between the intervention and control groups with the Intention-to-Treat analysis are summarized in Table 2. Significant differences were found in all outcome measures except the self-rating of quality of life measure (Table 2). There was a significant reduction in the average stress score in the intervention group in comparison to that of the controls with mean scores of 6.7 (s.e. = 0.4) and 7.5 (s.e. = 0.5) for the intervention and control groups, respectively (*p* = 0.015). This represented a weighted mean reduction of about 1.0 units (s.e. = 0.4) on the stress scale. For burnout, of the three domains of the MBI, no statistically significant differences between groups were found in two, namely emotional exhaustion and depersonalization. But a significant difference was observed in the professional accomplishment domain (*p* = 0.035). Participants in the intervention groups scored higher in professional accomplishment than the controls with mean scores of 28.2 (s.e. = 0.8) and 27.7 (s.e. = 1.2), respectively. This represented an increase in the weighted mean score of 1.9 units (s.e. = 0.9) on the scale for the intervention group. In terms of Mental Health Literacy, significant differences between groups were observed in all domains except social distancing (Table 2). Significant increases in the weighted mean scores were observed in the intervention group for correct recognition of a mental problem, help-seeking, and stigmatization, in comparison to the controls with 0.2 (s.e. = 0.1), 0.9 (s.e. = 0.2), 1.8 (s.e. = 0.4), respectively. Since the response set of the stigmatization scale was presented in a reversed order with a higher score representing a lower stigmatization attitude, an increased score on the scale reflected a reduction of the stigmatization attitude.

**Table 2:** Mean (s.e.) of the outcome measures assessed at the completion of the intervention program by groups and results on comparisons.

**Outcome measures**	**Control** **(*n* = 227)**	**Intervention** **(*n* = 229)**	**Results on comparison^a^**
Burnout — emotional exhaustion	20.5 (1.0)	20.9 (0.6)	*p* = 0.872
Burnout — depersonalization	6.9 (0.6)	7.4 (0.3)	*p* = 0.689
Burnout — professional accomplishment	27.7 (1.2)	28.2 (0.8)	***p*** **=** ***0.035***
Stress	7.5 (0.5)	6.7 (0.4)	***p*** **=** ***0.015***
Quality of life (self-rating)	80.5 (1.6)	81.5 (0.8)	*p* = 0.375
MHL — correct recognition	3.2 (0.1)	3.4 (0.1)	***p*** **=** ***0.003***
MHL — help-seeking	11.9 (0.2)	12.9 (0.3)	***p** **<** **0.001***
MHL — stigmatization	24.5 (0.6)	26.3 (0.5)	***p** **<** **0.001***
MHL — distancing	12.3 (0.6)	11.9 (0.5)	*p* = 0.160

a *Adjusted for the clustering effect, age, education level, and baseline assessment of the outcome measure. Bold values represent significant results*.

## Discussion

This trial aimed to investigate the efficacy of the purposefully designed intervention program for enhancing the mental wellbeing and mental health literacy of workers in the workplace. The results obtained from comparisons of the outcome measures between the intervention and control groups were statistically significant in favor of the intervention group on most measures, except for the quality-of-life measure. These results provide some evidence to support the efficacy of the intervention program under investigation. In general, these results are consistent with those observed in the literature. In terms of workplace mental health intervention, this trial specifically examined the effects on mental health literacy apart from burnout and stress which are common mental health outcomes of workplace programs (35, 36). Randomized Control Trials that focus on multiple aspects, including mental health wellbeing, cognitive understanding, and attitudes toward mental health, are not widely found in the literature. This study could be considered exceptional in the field. For the null result of the quality-of-life measure, there would be many reasons. One possible reason is related to the nature of the instrument. As noted by the developers of the instrument that: “EQ-5D is a standardized measure of health status developed by the EuroQol Group in order to provide a simple, generic measure of health for clinical and economic appraisal (37).” It is mainly designed for assessing patients with reasonably good utility. Although it could also be used in the general population, the utility of the instrument may not be as good as in the patient population, particularly when applied to a group of healthy participants. Another possible reason may be related to the timing of the study when the community was greatly affected by the pandemic and the general quality of life of the entire population was on the downside. The intervention program, as a workplace preventive strategy, has been designed with a specific focus on addressing workplace issues. Hence, participants may respond more positively to the work-related outcome measures.

To echo the appeal of the WHO in the mental health action plan 2013–2020 that the workplace is an important venue for mental health education and promotion, this study has provided substantiating evidence in demonstrating such value. The study has also rendered support to the argument that a well-designed workplace mental health intervention program is efficacious in alleviating stress and burnout, both are precursors to more severe mental health illnesses. Further, such a program can also enhance the mental health literacy of workers, which is a protective factor against mental health problems. There would be many practical implications drawn from the results of this study; two are outstanding. The first is related to the contents of the intervention program and the other is the use of digital technologies for health advancement, particularly in mental health. As suggested by Awa et al., a well-designed workplace mental health intervention program should consist of both individual-directed and organization-directed components to address issues arising from both parties. Work is a significant part of daily life and workers spend a large amount of time in the workplace. The resultant effect is a reciprocal relationship between workers and the work environment. The experience and mental state of workers will affect the work environment, and in turn, the physical and non-physical work environment will affect workers. Hence, any workplace mental health intervention program should address both variables. This would result in a better chance of success in assisting employees and employers toward an improvement of the overall wellbeing. In terms of the use of digital technologies for health advancement, particularly for mental health, there are ample examples in the last few years. The COVID-19 pandemic has also stimulated and motivated a more rapid development and adoption of digital health (DHealth) (38). In the post-pandemic era, it is foreseeable that healthcare provision and health advancement, including education and promotion, will be largely driven by and gain benefits from DHealth.

As in all trials, there are strengths and limitations in the current study that have been identified. First, the representativeness of the sample would be ascertained. Although participants were not recruited randomly from the working population, they were recruited by the Human Resources departments of five very large-size multinational corporates. These corporates are involved in a multitude of different businesses involving many different industries thus the work nature of their employees covers a wide range from manual labor to high-level executives. As a result, the sample reflects many sectors of the local working population and suggests the generalizability of the results. Second, the use of standardized and validated assessment instruments for all main variables of interest minimizes measurement and interpretation biases. Third, as shown by the results on comparisons of many baseline variables between the intervention and control groups, the randomization process is satisfactory further reducing the systematic basis of the trial. For the study design, this is a wait-listed cluster randomized controlled trial (CRCT) with the waiting control group not subjected to any placebo treatment. In terms of the design, a wait-list trial may not be as strong as a parallel-arm RCT with placebo treatment for the controls. Moreover, there would be a chance for treatment effect dilution for the controls to be exposed to the intervention program while they are still waiting, particularly for those participants who are working in the same environment. However, based on experience gained in previous trials utilizing a similar individualized Spaced-education approach for training, the chances for such dilution of treatment effect are slim and neglectable (39). Moreover, all participants involved in the study have been assigned a unique password for accessing the Web-based modules thus minimizing the risk of early exposure to the intervention materials of the controls. Another limitation identified is the missing values in some of the demographic variables. To reduce any bias in the effect estimation, Intention-To-Treat analysis was applied with the imputation of the missing values. As indicated, these missing values had exhibited a missing at random pattern upon examination. Hence, should there be any bases introduced by these missing values they are likely to be non-differential and exert a minimal effect on the strength of the effect estimates. Finally, this study has demonstrated the short-term effect of the intervention program. A follow-up study will present the results of the investigation of the long-term effect.

## Data Availability Statement

The datasets presented in this article are not readily available because permission by the funder is required to release the dataset. Should there be a request, permission could be sought through the institute's Research Office. Requests to access the datasets should be directed to LL, lawrence.lam@uts.edu.au.

## Ethics Statement

The studies involving human participants were reviewed and approved by institution ethics approval for the study was granted by the Human Research Ethics Committee of the Tung Wah College (Ethics Approval number: REC2018020). The patients/participants provided their written informed consent to participate in this study.

## Author Contributions

LL, ML, PW, and PR designed the study. LL obtained the funding, designed the statistical analysis plan, and will direct the data analyses. The Workplace Environment scan component of the intervention program was designed by LL and PW was responsible for the online and the face-to-face modules of the psychoeducation training. The data collection questionnaire was developed by LL, PW, and ML with the MHL scale translated and validated by LL with the permission of the original author. LL and PW authored the first draft of the study protocol to which ML and PR then contributed. All authors read and approved the final manuscript.

## Funding

This research was funded by the Health and Medical Research Fund, Food and Health Bureau, Hong Kong Government (Grant #02181028).

## Conflict of Interest

The authors declare that the research was conducted in the absence of any commercial or financial relationships that could be construed as a potential conflict of interest.

## Publisher's Note

All claims expressed in this article are solely those of the authors and do not necessarily represent those of their affiliated organizations, or those of the publisher, the editors and the reviewers. Any product that may be evaluated in this article, or claim that may be made by its manufacturer, is not guaranteed or endorsed by the publisher.
